# Reduced Motor Cortex Activity during Movement Preparation following a Period of Motor Skill Practice

**DOI:** 10.1371/journal.pone.0051886

**Published:** 2012-12-14

**Authors:** David J. Wright, Paul Holmes, Francesco Di Russo, Michela Loporto, Dave Smith

**Affiliations:** 1 Institute for Performance Research, Manchester Metropolitan University, Manchester, United Kingdom; 2 Department of Human Movement, Social and Health Sciences, University of Rome “Foro Italico”, Rome, Italy; 3 Neuropsychology Centre, Santa Lucia Foundation, IRCCS, Rome, Italy; Katholieke Universiteit Leuven, Belgium

## Abstract

Experts in a skill produce movement-related cortical potentials (MRCPs) of smaller amplitude and later onset than novices. This may indicate that, following long-term training, experts require less effort to plan motor skill performance. However, no longitudinal evidence exists to support this claim. To address this, EEG was used to study the effect of motor skill training on cortical activity related to motor planning. Ten non-musicians took part in a 5-week training study learning to play guitar. At week 1, the MRCP was recorded from motor areas whilst participants played the G Major scale. Following a period of practice of the scale, the MRCP was recorded again at week 5. Results showed that the amplitude of the later pre-movement components were smaller at week 5 compared to week 1. This may indicate that, following training, less activity at motor cortex sites is involved in motor skill preparation. This supports claims for a more efficient motor preparation following motor skill training.

## Introduction

Prior to performance of a voluntary movement there is a negative increase in the low-frequency electrical activity of the brain, called the movement-related cortical potential (MRCP). First discovered by Kornhuber and Deecke [1], the pre-movement MRCP consists of: (i) the Bereitschaftspotential (BP), an initial gradual increase in negativity that begins about two seconds prior to movement onset, is maximal over centro-parietal areas and widely distributed across the scalp [2]; (ii) the Negative Slope (NS’), a steeper gradient increase in negativity, occurring about half a second prior to movement onset and localized to the primary motor cortex and lateral pre-motor cortex [2]; and (iii) the Motor Potential (MP), a negative peak that occurs concomitant to movement onset and is localized to the contralateral primary motor cortex and sensorimotor cortex [3]. These pre-movement components of the MRCP are known to vary, depending on the physical and psychological characteristics of the forthcoming movement [4]. As such, it is widely accepted that these components of the MRCP reflect the cortical activity involved in motor preparation [5].

In recent years, several researchers have used the MRCP to study motor skill learning. Generally experimenters have examined differences in the amplitudes and onset times of the MRCP between a group of skilled performers and a control group of novices who have limited experience in a given skill (e.g., [6]–[10]). Collectively, these studies have reported differences in MRCP components that include smaller amplitudes and later onset in the skilled performers compared to the novices. These differences have consistently been interpreted as an indication that skilled performers require ‘less effort’ than novices during movement preparation. Long-term training or practice by the skilled performers is typically attributed as the reason for this difference. Similar cross-sectional studies using functional magnetic resonance imaging (fMRI) have also reported reduced activity in a variety of movement-related brain areas in skilled musicians compared to novices during piano-based tasks, and attributed the differences to training by the skilled group (e.g., [11]–[14]).

Although these studies have provided some interesting insights into the cortical processes that may be involved in motor skill learning, it is problematic to claim that the reported differences are due to long-term training based solely on such expert-novice comparison studies. To adequately demonstrate that the results are learning-related, a change in performance or activity must be observed over a period of time, and as a result of practice or experience [15]. Therefore, longitudinal studies that assess possible changes in cortical activity in the same participants over the course of a training program are required to support the claims made in cross-sectional studies [16], [17]. This is necessary, as the differences reported in cross-sectional studies may not necessarily be due to long-term training in the expert group. It is perhaps equally possible that the differences reported in cross-sectional studies were inherent to the performers, as opposed to an adaptation resulting from training [18]. For example, highly skilled performers may have an innate predisposition that requires fewer cortical resources when preparing to perform certain motor skills. Such a predisposition may make them more likely to excel at the skill, continue to train in that skill, and reach an expert level. Longitudinal studies, plotting potential changes associated with skill learning over weeks and months are, therefore, warranted.

Several attempts have been made to study the effects of learning on cortical activity by plotting changes in the MRCP that are associated with repetitive practice of a movement, but only over the course of a single testing session (e.g., [19], [20]). These studies reported smaller amplitude MRCPs in later blocks of trials compared to earlier blocks. This led the authors to conclude that following practice, the amount of effort required to plan and perform a motor skill is reduced. Although interesting, we propose that these changes more likely reflect the effects of short-term repetitive practice, rather than actual learning. Additionally, both these studies investigated changes in cortical activity related to the practice of either simple button pressing [20] or wrist and finger flexion-extension sequences [19], rather than more ecologically valid motor skills, such as playing a musical instrument.

To date, no studies have considered changes in the MRCP associated with a period of ecologically valid motor skill training, and using a longitudinal design. This has led to a call for future studies to investigate changes in cortical activity, in a more ecologically valid way, over a longer period of weeks or months [16], [17]. This study aimed to address this gap in the literature. In pilot testing for the study we first confirmed the reproducibility and stability of the MRCP on separate day testing sessions. Following this we plotted possible changes in the MRCP over the course of a 5-week training program on the guitar. We hypothesized that, following a period of training, participants would show MRCPs of smaller amplitude and later onset at the end of the training program compared to the start.

## Methods

### Participants

Eight participants (4 male, 4 female; mean age = 23.51 years ±9.47) took part in the pilot testing to confirm the reproducibility and stability of the MRCP. Ten non-musicians (5 male, 5 female; mean age = 26 years±9.35) with no prior experience of the playing the guitar or any other musical instrument participated in the training study. Different participants took part in the two different phases of the study. All participants were right handed as assessed by the Edinburgh Handedness Inventory [21]. All participants gave their written informed consent to take part in the study, which was conducted in accordance with the Declaration of Helsinki. The experimental procedures were granted ethical approval by the Manchester Metropolitan University’s Exercise and Sport Science Departmental Ethics Committee (ethical approval number: 30.11.09i).

### Procedure

#### Pilot testing

To confirm the reproducibility and stability of the MRCP within participants over separate day testing sessions, the MRCP was recorded on two separate occasions as participants performed 100 repetitions of a self-paced button pressing task with their right index finger. The mean interval between the two testing sessions was 30.12 days (±6.76). Establishing reproducibility and stability of the MRCP was necessary to ensure that any changes reported in the subsequent training study could be accurately attributed to the training undertaken by the participants, and were not simply the result of variability in the MRCP. To analyze the data, amplitude values from all electrode sites were averaged to provide mean amplitude values at pre- and post-test. A point-by-point paired samples *t* test was then used to compare differences in the amplitude of the MRCPs recorded at pre- and post-test within a time window of 1500 ms prior to movement onset to 500 ms post movement onset.

#### Training study

**Figure 1:**
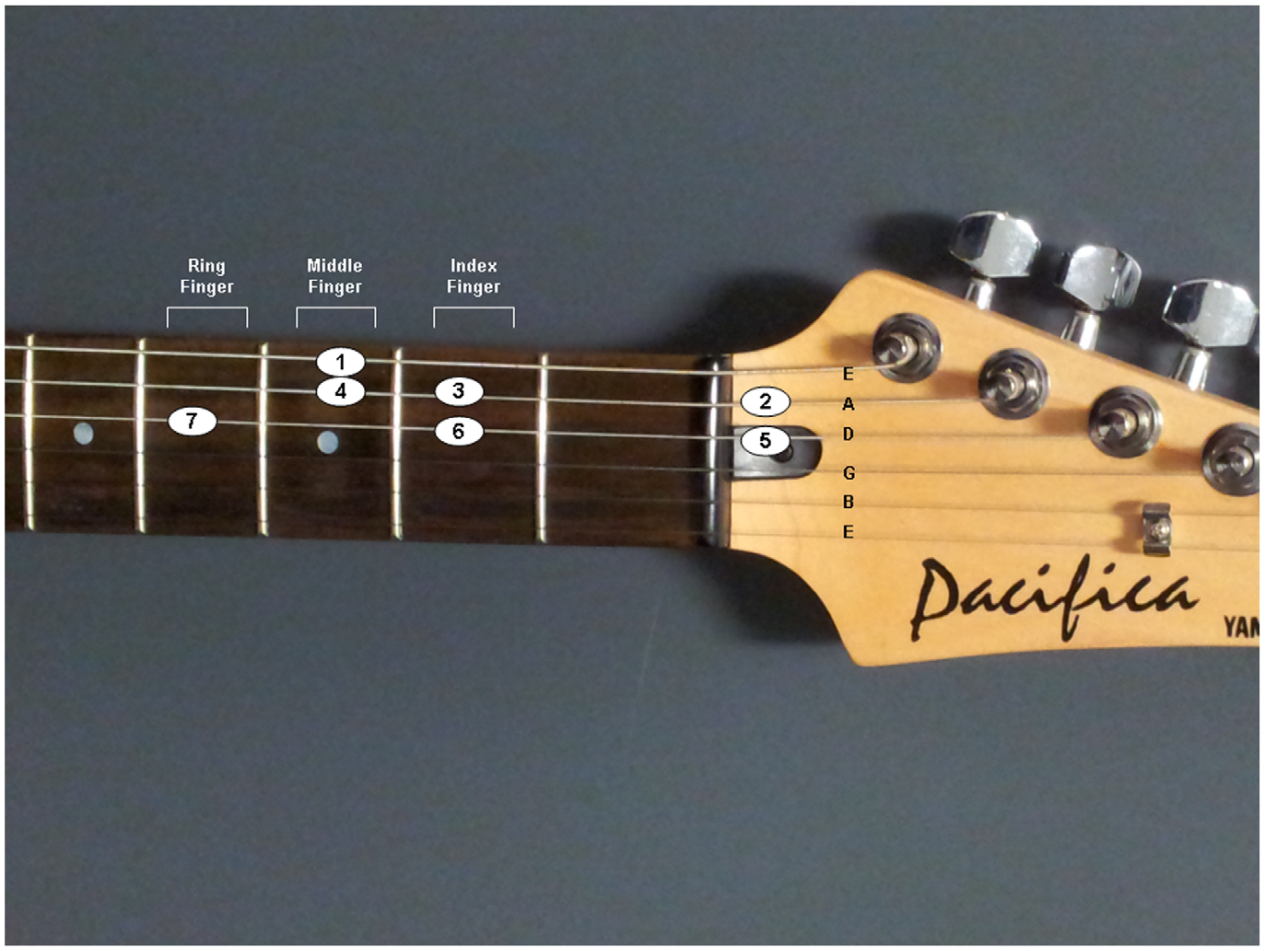
The first seven notes of the G Major scale as played on the guitar.

**Figure 2:**
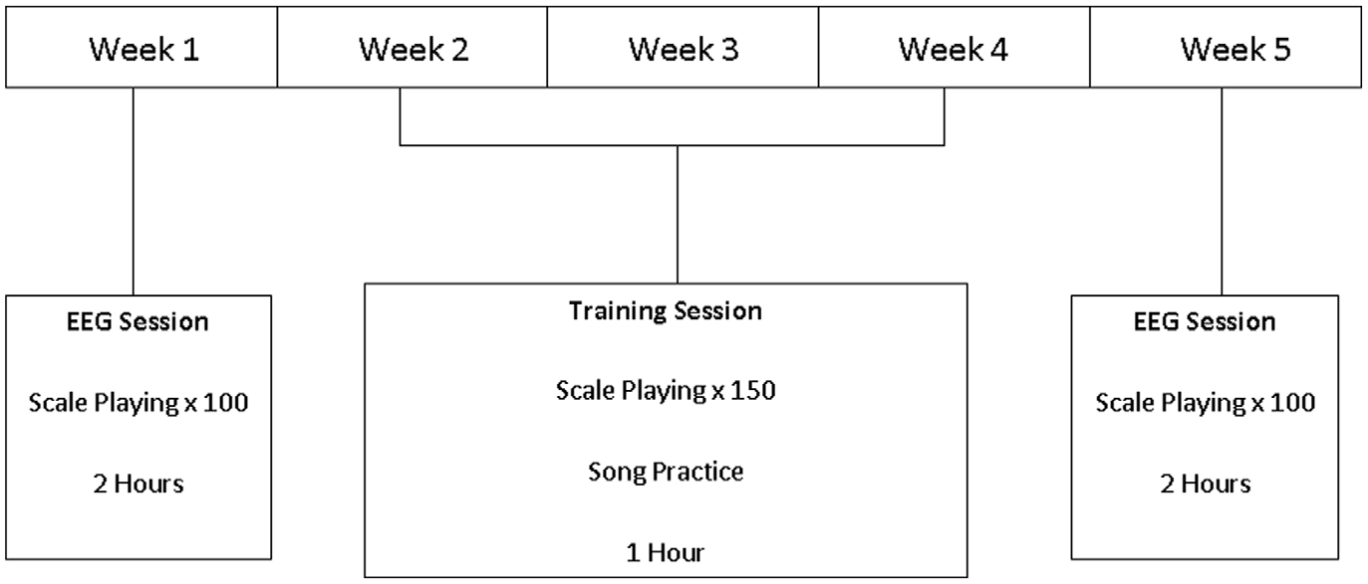
Protocol for the training study.

Participants took part in a five-week training program learning to play the guitar. During this period, they were required to attend one testing session per week. At week 1, they were provided with 15 minutes instruction on how to play the G Major scale on the guitar. Following this, they were seated and instructed to play 100 repetitions of the first seven notes of the G Major scale (see Figure 1) on a Yamaha Pacifica 112V electric guitar, whilst electroencephalography (EEG) was recorded. Participants were instructed to leave approximately 10 seconds between each repetition of the scale. To reduce artifacts in the EEG recording, participants were also encouraged to avoid blinking or making any movements immediately before beginning a repetition. The numbered circles depicted in Figure 1 indicate the ascending order in which the notes were played. Participants played notes on the second fret with their index finger, third fret with their middle finger and fourth fret with their ring finger. A metronome ran continuously throughout this period at 100 beats per minute (bpm), and participants were instructed to try to play the scale in time with the metronome. The G Major scale played at a tempo of 100 bpm was selected, as it is a ‘Rockschool’ rock and pop music examination board Grade 2 assessment piece [22]. Based on consultation with a ‘Rockschool’ assessor, it was expected that the participants would be able to play a Grade 2 scale with some practice. At weeks 2–4 participants received an individual one hour guitar lesson. Each lesson was split into three parts. First, participants spent 15 minutes practicing the G Major scale in time with the metronome at 100 bpm. Second, participants spent 30 minutes practicing some simple songs on the guitar. Participants then spent the final 15 minutes of the lesson performing further practice of the scale in time with the metronome. During each 15-minute practice period, participants performed 75 repetitions of the scale, resulting in a total of 150 repetitions per lesson. The purpose of the song practice section of the lessons was to make the lessons more enjoyable for the participants and keep them motivated, in an attempt to reduce participant dropout. At week 5, participants returned for a final EEG testing session, with the same procedure as week 1. The protocol for this experiment is shown in Figure 2.

At weeks 1 and 5, after the 100 trials were performed alongside the EEG recording, the guitar was then connected into an Apple Mac Mini computer (Apple, Cupertino, CA, USA) and participants were asked to perform an additional 20 repetitions of the scale, again at 100 bpm. These performances were recorded using Logic Express (version 9) software (Apple, Cupertino, CA, USA) and assessed offline. Performances were assessed in terms of the participants’ ability to play the scale in time with the metronome. Using the software it was possible to measure the millisecond difference between the beat of the metronome and the note being played. It was not possible to assess performance concurrent with the EEG recording since connecting the guitar into the performance recording equipment introduced noise into the EEG recording.

### Electrophysiological Recording

EEG was recorded during the pilot testing and at weeks 1 and 5 of the training study from scalp electrodes located over the motor and premotor cortex. Six, 6 mm diameter, silver/silver-chloride electrodes were placed at sites FC3, FCz, FC4, C3, Cz and C4 according to the International 10–10 system of electrode placement [23]. Electro-oculography (EOG) was recorded from electrodes placed below and adjacent to the left eye to monitor horizontal (HEOG) and vertical (VEOG) eye-movements. All electrodes were referenced to linked mastoids and a ground electrode was placed at site Fpz. Prior to attaching the electrodes, the scalp sites were abraded with NuPrep skin preparation paste (DO Weaver, Aurora, CO, USA). Electrodes were then secured to the scalp using Ten-20 conductive and adhesive EEG paste (DO Weaver, Aurora, CO, USA). Following electrode attachment participants had a 45-minute rest period before testing began to minimize signal drift. Electrode impedances were checked and kept homogenous at, or below, 5 kΩ throughout the experiment. The EEG and EOG were recorded with a gain of 1000 and an A/D sampling rate of 1000 Hz, using Scan 4.3 software and a NeuroScan Synamps amplifier (Compumedics Neuroscan, Charlotte, NC, USA). Cortical channels were recorded with a 0–30 Hz bandpass filter, whilst EOG channels were recorded with a 0.15–30 Hz bandpass filter.

### Data Analysis

During the training study the movement trials were averaged and referenced to the point of movement onset. Movement onset was defined as the point at which the bottom E string was pressed against the fret board to play the first note of the scale. This was recorded using a thin electrode attached to the neck of the guitar behind the strings at the third fret, which was connected into a ‘movement onset’ channel in the EEG amplifier. When the bottom E string was pressed at the third fret to play the first note of the scale, the string made contact with the electrode and caused a sharp deflection to occur on the movement onset channel in the EEG recording. Digital markers were then inserted onto the EEG trace at points where the sharp deflection on the movement onset channel exceeded 50 µV in amplitude.

Prior to analysis, an automatic eye-movement rejection was applied to the raw data. All sections of the EEG recording that contained artifacts in excess of 50 µV on either the VEOG or HEOG were removed from the recording. On average, this resulted in the removal of 16 trials (±11.04) at week 1, and 17 trials (±12.22) at week 5 from each participant. The EEG recording was then filtered offline using a 0–5 Hz bandpass filter to remove the higher frequency signals from the trace. Following this, the data were split into epochs of three seconds around the movement onset marker. Each epoch contained 2500 ms of data prior to movement onset and 500 ms of post-movement data. The epochs were then averaged together to produce the MRCP. Finally, prior to analysis, the MRCP microvolt values were converted into *z*-scores and referenced to a baseline period of 2500 to 2000 ms prior to movement onset. The purpose of this was to normalize the data and remove variability in the baseline amplitudes between participants.

For statistical analysis, the mean amplitudes and onset times of the BP and NS’, together with the peak value of the MP were obtained from the MRCP data at all electrode sites. Following the methods of previous MRCP experiments [7], [9], [10], onset times for the BP and the NS’ components were established by visual inspection by the first author and these values were then subsequently confirmed independently by the fourth author. Using Scan 4.3 software it was possible to place a cursor marker at the observed onset of the BP and NS’, and obtain an exact millisecond value at each cursor placement. The criteria for selecting these onset times were based upon the descriptions of each component provided by Shibasaki and Hallett [5]. The onset of the BP was identified as the observed onset of a gradual increase in the negativity of the EEG that began approximately 1800–2000 ms prior to movement onset. The onset of the NS’ was then identified as the observed onset of a much sharper increase in the negativity of the EEG that occurred around 500–750 ms prior to movement onset. Amplitude values for the BP and the NS’ components were based around their onset times. The BP amplitude was taken as the mean amplitude from the time of the BP onset to the time of NS’ onset. Similarly, the NS’ amplitude was taken as the mean amplitude from the time of NS’ onset to the peak of the MP. The MP amplitude was taken as the peak amplitude of the pre-movement MRCP, immediately prior to movement onset. Statistical analysis was performed using the SPSS for Windows 16.0 statistical package. The mean amplitudes and onset times of the BP and NS’ components of the MRCP, together with the peak MP values were submitted to separate 2 time (week 1, week 5)×6 electrode (FC3, FCz, FC4, C3, Cz, C4) repeated measures analysis of variance (ANOVA). Where Mauchley’s test indicated that sphericity had been violated, the degrees of freedom were corrected using the Huynh-Feldt method. Post-hoc interpretations were made using Duncan’s multiple range tests. The performance measure was submitted to a paired samples *t* test. All significant effects were reported at an alpha value of *p*<.05 and adjusted where necessary. Effect sizes are reported as partial eta squared (η^2^_ρ_).

## Results

### Pilot Study Data

**Figure 3:**
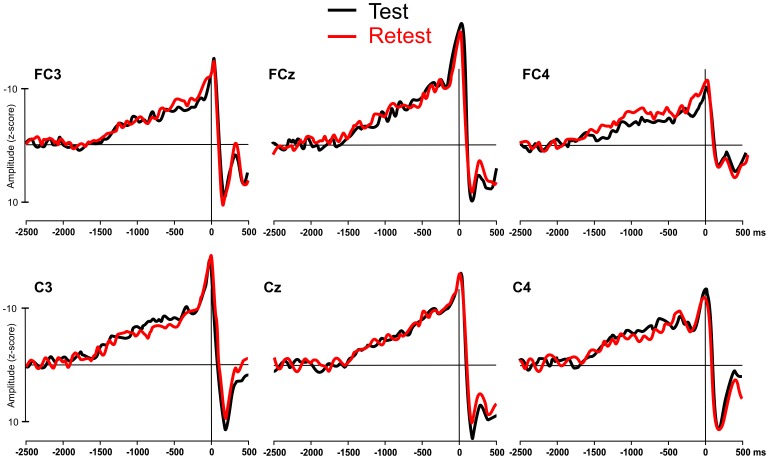
Low variability of the MRCP waveforms recorded from the motor cortex during a button pressing task on two occasions, approximately 30 days apart.

Clearly visible MRCPs were recorded from all participants at all six electrode sites at both the pre- and post-test recording sessions in the pilot study. Visual inspection indicated little difference between the pre- and post-test MRCP waveforms in either the amplitude or onset times of the pre-movement components (see Figure 3). This similarity was confirmed by a point-by-point paired samples *t* test which indicated no differences in the amplitude of the pre- and post-test MRCPs at any time point between 1500 ms prior to movement onset and 500 ms post movement onset (t = 1.84, df = 7, *p = *.11). This finding suggests that the MRCP is reproducible and stable when recorded from the same participants on separate days.

### Training Study Data

**Figure 4:**
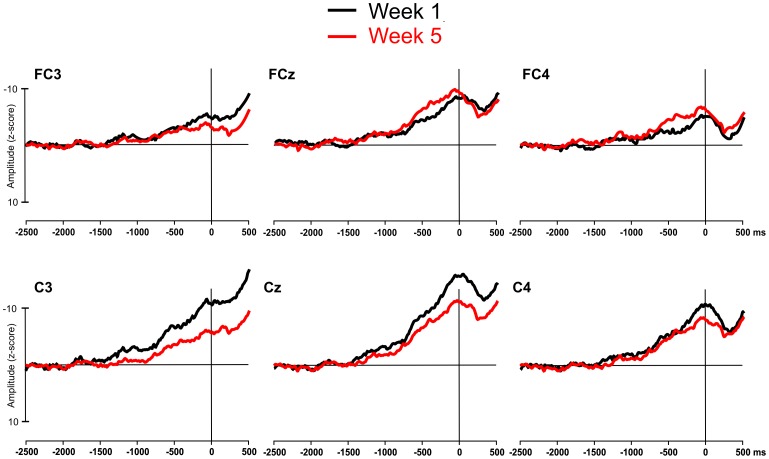
MRCP waveforms recorded from the motor cortex during performance of the scale on the guitar at week 1 and week 5 of the training study.

**Figure 5:**
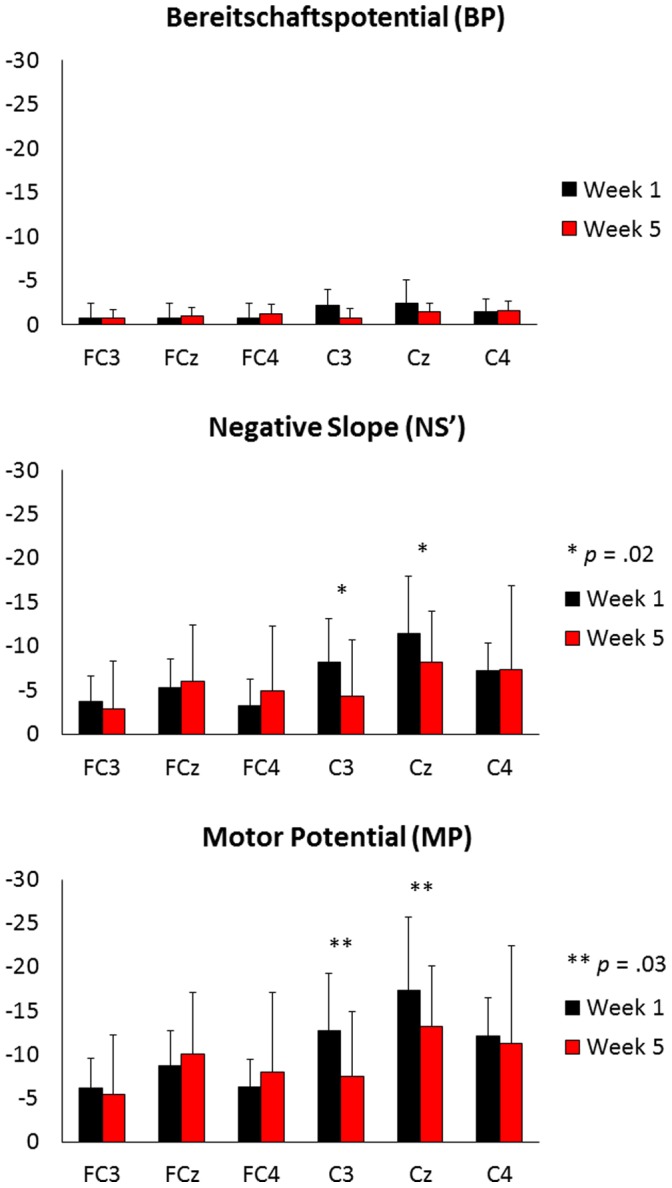
Mean amplitude values of the MRCPs components recorded at week 1 (black) and week 5 (red).

A clear MRCP was evident in all participants at all six electrodes sites at both week 1 and week 5. The MRCP waveforms from each electrode, recorded at week 1 and week 5, are displayed in Figure 4. The mean amplitudes and onset times of the individual components of the MRCP are shown in Figure 5 and Table 1, respectively.

**Table 1:** Mean (± SD) onset times (ms) for BP and NS’ components of the MRCP at week 1 and week 5, together with *p* values from the ANOVA analysis.

	Week 1	Week 5	*p* value
BP Onset (ms)	−1804 (±245)	−1856 (±297)	.64
NS’ Onset (ms)	−691 (±193)	−737 (±195)	.63

A separate ANOVA was conducted for each component of the MRCP.

#### Bereitschaftspotential (BP)

At both week 1 and week 5, the BP initiated around 1800 ms prior to movement onset and increased gradually until around 700 ms prior to movement onset. The onset times of the BP at week 1 and week 5 are shown in Table 1. The repeated measures ANOVA for the BP onset time revealed no significant main effect of time (F_1,9_ = 0.24, *p* = .64, η^2^_ρ = _.03), or electrode (F_5,45_ = 0.292, *p* = .79, η^2^_ρ = _.031). In addition, there was no significant time×electrode interaction (F_5,45_ = 0.77, *p* = .54, η^2^_ρ = _.08).

The amplitude of the BP was taken as the mean amplitude between BP onset and NS’ onset. The mean *z*-score amplitude for the BP was −1.36 (±1.91) at week 1, compared to −1.1 (±3.25) at week 5. The repeated measures ANOVA revealed no significant main effect of time (F_1,9_ = 0.053, *p = *.82, η^2^_ρ = _.006), or electrode (F_5,45_ = 2.15, *p = *.08, η^2^_ρ = _.19). Additionally, for the BP amplitude, there was no significant time×electrode interaction (F_5,45_ = 1.73, *p* = .15, η^2^_ρ = _.16).

#### Negative Slope (NS’)

The onset times of the NS’ at week 1 and week 5 are shown in Table 1. The repeated measures ANOVA for the NS’ onset time indicated that there was no significant main effect of time (F_1,9_ = 0.25, *p* = .63, η^2^_ρ = _.03), or electrode (F_5,45_ = 0.38, *p = *.79, η^2^_ρ = _.041). In addition, there was no significant time×electrode interaction (F_5,45_ = 0.64, *p* = .67, η^2^_ρ = _.07).

The amplitude of the NS’ was taken as the mean amplitude from NS’ onset to the peak of the MP. The mean *z*-score amplitude for the NS’ was −6.5 (±4.88) at week 1, compared to −5.62 (±6.86) at week 5. The repeated measures ANOVA revealed no significant main effect of time (F_1,9_ = 0.2, *p* = .67, η^2^_ρ = _.022). There was however a significant main effect of electrode (F_5,45_ = 8.31, *p*<.001, η^2^_ρ = _.48). The post-hoc comparison revealed that the NS’ amplitude at Cz was larger than at FC3 and FC4, whilst the amplitude at C4 was larger than at FC3. In addition, there was a significant time×electrode interaction (F_4.8,43.3_ = 2.93, *p* = .02, η^2^_ρ = _.25). The post-hoc analysis indicated that the amplitude of the NS’ was smaller at week 5, compared to week 1, at sites C3 and Cz.

#### Motor Potential (MP)

The amplitude of the MP was taken as the peak of the MRCP, corresponding to the maximum negative peak immediately prior to movement onset. The mean *z*-score amplitude for the MP at week 1 was −10.58 (±6.49), compared to −9.25 (±8.28) at week 5. The repeated measures ANOVA revealed that there was no significant main effect of time (F_1,9_ = 0.419, *p* = .54, η^2^_ρ = _.049). There was however a significant main effect of electrode (F_5,45_ = 10.49, *p*<.001, η^2^_ρ = _.54). The post-hoc comparison showed that the amplitude of the MP at Cz was significantly larger than at FC3, FCz, FC4, and C3. Similarly, the amplitude of the MP at C4 was larger than at FC3. In addition, there was a significant time×electrode interaction (F_4,36.2_ = 2.98, *p* = .03, η^2^_ρ = _.25). The post-hoc analysis indicated that the amplitude of the MP was smaller at week 5, compared to week 1, at sites C3 and Cz.

### Combined Data

To verify that the differences reported between week 1 and week 5 were due to the training undertaken by the participants, data from any components of the MRCP that were found to change significantly from week 1 to week 5, were compared against the pilot study data (see [24] for rationale). The training study indicated significant decreases in the amplitude of the NS’ and MP components of the MRCP at electrode sites C3 and Cz. Two separate 2 group (control, training) ×2 electrode (Cz, C3) ×2 time (pre, post) ANOVAs were therefore conducted, one for the NS’ and one for the MP. This resulted in a significant group×time interaction for both the NS’ (F_1,16_ = 4.56, *p = *.05, η^2^_ρ = _.22) and the MP (F_1,16_ = 4.56, *p = *.04, η^2^_ρ = _.25). This indicates that the amplitude of the NS’ and MP at sites C3 and Cz were reduced from week 1 to week 5 only in the training group, and not the control participants from the pilot study.

### Performance Data

At week 1, the participants performed the scale with a mean of 749 ms (±1074) error between the beat of the metronome and the note being played. At week 5, after several weeks of practicing the scale, the participants performed the scale with a mean of 273 ms (±582) error between the beat of the metronome and the note being played. A paired samples *t* test confirmed that the participants’ ability to play in time with the metronome had significantly improved over the course of the training program (t = 2.219, df = 9, *p* = .05).

## Discussion

The aim of this study was to investigate possible changes in the pre-movement components of the MRCP as a result of learning to play a scale on the guitar over a period of five weeks. The objective of this was to verify the claims made in previous cross-sectional MRCP studies (e.g., [6]–[10]), that following a period of training reduced activity is required by the premotor and motor cortices to plan and prepare to perform a skilled action. To the best of our knowledge, this represents the first attempt to study changes in the MRCP associated with ecologically valid motor skill training over a longitudinal period. In pilot testing we recorded the MRCP from participants as they performed repetitions of a simple button pressing task on two occasions around 30 days apart. Our analysis showed no differences in the amplitude of the MRCP between the two testing sessions. This confirmed that the MRCP is stable and reproducible within participants over separate days. A separate group of participants then took part in a five-week training program, where they learned and practiced playing a scale on the guitar. We found no change in the onset times of the BP and NS’ components as a result of the training program. However, although no change in amplitude was found for the BP, there was a significant decrease in the amplitude of both the NS’ and MP components at electrode sites C3 and Cz as a result of the 5-week training program. The combined analysis then confirmed the validity of these results by demonstrating that the reduction in NS’ and MP amplitude was only present at these electrode sites in the training participants, and not the control participants from the pilot study. A change in the amplitude of the MRCP is thought to reflect a change in the amount of effort involved in movement preparation [19]. Therefore, the decrease in NS’ and MP amplitude over the course of the training program may indicate that less effort is required during motor preparation by certain areas of the motor cortex, as a result of learning the skill. The reduction in cortical activity over the course of the training program in this study was also accompanied by a significant improvement in performance. These results may therefore indicate that, as an individual becomes more competent in a motor skill, fewer cortical resources are required to be devoted to its planning and performance.

These findings are mostly consistent with our hypothesis. The results support the claims made in both MRCP studies [6]–[10], [19], [20] and fMRI studies [11]–[14], that following long-term training in a particular skill, fewer cortical resources are required to plan and perform that skill. This result is also consistent with the concept of ‘neural efficiency’ following motor skill learning. According to this concept, individuals who perform a skill to a high standard are likely to have a more efficient cortical functioning when performing that skill, compared to individuals who perform to a lower standard [25], [26]. In the current study improvements in performance over a five-week period were accompanied by a reduced cortical processing in certain areas of the motor cortex. Although the sample used in this study was relatively small, the significant findings make an important contribution to the literature as, to the best of our knowledge, these results represent the first longitudinal evidence in support of the concept of neural efficiency. The claim that these results are learning-related is backed up by the pilot testing data we reported. As the MRCP is stable and reproducible within participants over separate days (see Figure 3), we can be fairly certain that the reduction in the amplitude of the NS’ and MP we reported between weeks 1 and 5 are learning-related changes, as opposed to being the effect of variability in the MRCP measurement or the effect of participant habituation with the recording procedure due to the repeated measures design.

Although our findings offer support for the concept of neural efficiency following motor skill learning, an alternative explanation could be that the reduced activity in the motor cortex is accompanied by an increase in activity in other movement-related brain regions, such as the cerebellum or basal ganglia. For example, the findings of Jueptner et al. [27], [28] suggest that the motor cortex is initially involved in skill learning but as the skill becomes more well-learned the motor cortex becomes less active and the cerebellum ‘takes over’ control of the movement. However, as we only recorded our EEG data from six electrodes located over the pre-motor and motor cortices it is not possible for us to speculate further on this issue. We would therefore encourage researchers in future experiments to investigate changes in cortical activity associated with motor skill learning to use a larger and more dense electrode montage than used in the current study. It would also be particularly worthwhile for future studies to combine EEG and fMRI techniques. As fMRI is able to record activity from sub-cortical regions such as the cerebellum and basal ganglia it would be possible to establish whether motor skill learning produces a neural efficiency of all motor regions or whether the reduced activity we reported in the motor cortex is indicative of a shift in the locus of control to other movement-related brain regions.

The reduction in the amplitude of the NS’ and MP was significant at electrode sites C3 and Cz following the five-week training program. Electrode site Cz is approximately located over the supplementary motor area (SMA); a medial frontal area of the brain involved in motor planning and bimanual control [29]. It is also the area of the brain where the early components of the MRCP are generated and of maximal amplitude [5]. Due to the bimanual nature of the task, it is likely that the SMA was involved in both the planning and the performance of the task across all weeks. The reduction in the NS’ and the MP amplitude found at Cz was, therefore, expected. The reduction in amplitude found at site C3 but not C4 could, in part, be due to the different hemispheric contribution in the bimanual task. Electrode site C3 is located over the motor representation for the right hand, whilst C4 is located over the motor representation for the left hand. In the scale-playing task, the action performed by the right hand (plucking the strings with a plectrum) is arguably simpler than that performed by the left (fingering the notes along the fretboard). As such, participants may have learnt the right hand part of the task more easily than the left, promoting the reduction in amplitude at C3, but not C4. With the small number of electrodes used in this study, however, it is not possible to speculate further on this issue. Future research, using a more dense electrode montage, could provide a better explanation as to the topography of the learning process. It should be noted that the MP, which reflects contralateral activity in primary motor cortex, is slightly larger (especially in week 5) over the right hemisphere sites, consistent with the prediction of a greater contribution made by the left hand.

In addition to the reduction in amplitude of the NS’ and the MP components, and based on the cross-sectional skill learning MRCP studies (e.g., [6]–[9]), we had also anticipated a reduction in the amplitude of the BP. However, no change in the amplitude of the BP was found. This could be due to the presence of the metronome used in this study to control movement tempo. The metronome ran continuously throughout the experiment at 100 bpm and, whilst participants were free to begin each repetition of the scale at a time of their choosing, they were instructed to try to keep their playing in time with the metronome. The movement decision to begin each repetition could, therefore, have been influenced by the metronome rather than being a self-initiated decision. In a study by Di Russo et al. [30], the authors reported that when flexion movements of the index finger were self-initiated by the participant, both the BP and NS’ components were present, however the BP component was absent when the same movement was externally triggered by a tone. The presence of the metronome in the current study could have acted partially as an external trigger to begin playing the scale. Therefore, although the BP component was present in this study, the presence of the metronome may have reduced the amplitude of the BP and contributed to the lack of change in the BP over the five-week training program.

In relation to the onset times of the MRCP components, previous expert-novice cross-sectional studies have reported later onset times for both the BP and NS’ in expert performers compared to novices (e.g., [6]–[9]). This has been interpreted to indicate a more efficient motor preparation. In a similar way to the amplitude differences between expert and novice performers, researchers generally attribute the onset time differences to long-term training by the expert group. Consequently, we predicted that there may have been a change in the BP and NS’ onset times across the five-week training program, with onset times at week 5 predicted to occur later than at week 1. However, contrary to our predictions, we found no differences in the onset times of either component between week 1 and week 5. It is possible that the time-scale required to see differences in MRCP component onset times is different to that for amplitude changes. Generally, in the expert-novice cross-sectional studies, participants with many years of training in a particular skill are compared to a group of novices with no prior experience in that skill. As such, the differences seen in the cross-sectional studies are likely to show the effects of long-term learning over many years, as opposed to weeks. Therefore, although a reduction in amplitude was evident after five weeks of training, it may take longer for a change in onset latency to occur. We propose that if the training program had been extended further, changes in MRCP onset latency may have been evident.

Although this line of work is still at a relatively early stage, the potential applications of a greater understanding of the cortical changes involved in motor learning are important and worth emphasizing. For example, there may be useful applications for the treatment of disorders of the motor system, such as Parkinson’s disease, and for stroke rehabilitation. In the case of stroke rehabilitation, for example, movement therapies are often administered with the intention of promoting (re)learning of movements through neuronal reorganization in the affected cortical areas [31]. A more comprehensive understanding of the cortical changes that occur with learning may therefore aid the design and administration of such therapies. We therefore strongly recommend that researchers explore these changes in greater detail, using larger sample sizes, more dense electrode montages, longer time periods, and techniques such as fMRI and transcranial magnetic stimulation.

To conclude, we have shown that following a period of motor skill training, and as an individual becomes more competent in performing a skill, the amount of effort required during motor planning of that skill is reduced in specific areas of the motor cortex. This finding is consistent with the concept of neural efficiency following motor skill learning [25], [26]. We believe this to be the first study to demonstrate this phenomenon during the learning of an ecologically valid motor skill over a longitudinal period. Future studies, combining fMRI and EEG with larger and more dense electrode montages and investigating changes in activity over a longer learning period should further explore the topography of this phenomenon.
